# Primary Adrenal Insufficiency in Acute Progressive Systemic Inflammation Accompanied by Latent Tuberculosis: A Case Report

**DOI:** 10.7759/cureus.48695

**Published:** 2023-11-12

**Authors:** Kasumi Nishikawa, Mizuki Nitta, Shoma Tanaka, Chiaki Sano, Ryuichi Ohta

**Affiliations:** 1 Family Medicine, Shimane University Hospital, Izumo, JPN; 2 Family Medicine, Shimane University Medical School, Izumo, JPN; 3 Community Medicine Management, Shimane University Faculty of Medicine, Izumo, JPN; 4 Community Care, Unnan City Hospital, Shimane, JPN

**Keywords:** community hospital, japan, family medicine, general medicine, eosinophils, latent tuberculosis, primary adrenal insufficiency, adrenal insufficiency

## Abstract

A 64-year-old man presented with general malaise, edema, and other nonspecific symptoms, prompting extensive diagnostic evaluation. The patient’s early morning cortisol and adrenocorticotropic hormone levels were consistent with primary adrenal insufficiency without evident secondary or tertiary causes on magnetic resonance imaging. The interferon gamma release assay (T-SPOT^®^) was positive, suggesting latent tuberculosis, although there were no signs of active tuberculosis. The potential of extrapulmonary tuberculosis as a causative factor for adrenal insufficiency was explored but remained unconfirmed on contrast-enhanced computed tomography. Eosinophilia was detected, suggesting a link between adrenal insufficiency and the occurrence of atopic dermatitis. This case underscores the multifaceted nature of adrenal insufficiency and its potential associations. While autoimmune conditions are commonly associated with adrenal insufficiency, infectious diseases (e.g., tuberculosis) can also be contributing factors. Eosinophilia further indicates the likelihood of coexisting allergic or atopic conditions, particularly adrenal dysfunction. Although not dominant, the presence of latent tuberculosis can cause severe complications, including adrenal insufficiency, highlighting the requirement of vigilant monitoring. Clinicians are advised to consider adrenal insufficiency in the differential diagnosis of patients with generalized symptoms and perform comprehensive evaluations, including cortisol level assessment and tuberculosis screening.

## Introduction

Adrenal insufficiency presents with various symptoms associated with decreased secretion of glucocorticoids, mineralocorticoids, and androgens caused by the destruction or lack of stimulation of the adrenal glands. It is classified into three categories based on the site of damage in the secretory mechanism: primary (adrenal gland), secondary (pituitary gland), and tertiary (hypothalamus). Primary adrenal insufficiency is rare, with a prevalence of approximately 87-221 per million in European countries [1-3] and approximately four per million in Korea, which neighbors Japan [4]. The prevalence of secondary adrenal insufficiency is approximately 150-280 per million [5]. Tertiary adrenal insufficiency, the most common form of adrenal insufficiency, is caused by long-term steroid administration [6]. With age, patients may develop various complications, and the causes of adrenal insufficiency should be comprehensively analyzed.

Latent tuberculosis can cause adrenal insufficiency and should be included in the differential diagnosis of adrenal insufficiency in elderly patients. Latent tuberculosis should also be distinguished from extrapulmonary tuberculosis. Furthermore, patients with adrenal insufficiency and extrapulmonary tuberculosis can present with various complaints, making the diagnosis difficult. Here, we report a case of primary adrenal insufficiency and latent tuberculosis in a 64-year-old man, detected on close examination of general malaise and edema. We discuss the diagnostic assessments for primary adrenal insufficiency in patients with various complaints and treatment options for latent tuberculosis based on a close examination of primary adrenal insufficiency.

## Case presentation

A 64-year-old man presented to a community hospital with chief complaints of decreased appetite and abdominal pain. He had anorexia for a month. Simultaneously, he experienced epigastric pain accompanied by progressive anorexia. He also experienced pain in both legs and had difficulty walking. Three weeks before, he had visited his local doctor because of a loss of appetite and had been treated with intravenous fluids. Loss of appetite was present for one day. From the morning of his visit, he had been noticeably listless with sluggish movements, which led to emergency department admission at our hospital. The patient's past medical histories were sleep apnea, hypertension, depression, dyslipidemia, chronic atrial fibrillation, reflux esophagitis, diabetes mellitus, glaucoma, and asthma. The drug history included escitalopram 10 mg, brotizolam 0.25 mg, mirtazapine 15 mg, montelukast 10 mg, esomeprazole 20 mg, and pravastatin 10 mg daily without any steroid-containing over-the-counter drugs.

His vital signs were temperature, 37.3°C; blood pressure, 135/68 mmHg; pulse rate, 63 beats/min; respiratory rate, 18 breaths/min; and SpO2, 94% on room air. He was alert to time, person, and place. A physical examination of the abdomen revealed tenderness in the pericardial fossa and right lower abdominal region, with no recurrent pain or pain from the liver. Examination of the extremities revealed edema, redness, heat, and tenderness in both the lower legs. In addition, numbness was noted in the area from the fingertips to the neck, except for the first and fourth toes bilaterally. Examination of the joints revealed heat and tenderness in both hands, shoulders, and thigh joints. Abdominal ultrasonography revealed no abnormalities. The blood gas analysis showed a PCO2 of 52.8 mmHg and a PO2 of 23.3 mmHg, indicating type II respiratory failure. Blood samples showed elevated lactate dehydrogenase levels, C-reactive protein levels, and eosinophil count (813/μL) and reduced total protein and albumin levels (Table 1).

**Table 1:** Initial laboratory data for the patient eGFR: estimated glomerular filtration rate; CRP: C-reactive protein; TSH: thyroid-stimulating hormone; Ig: immunoglobulin; SARS-CoV-2: severe acute respiratory syndrome coronavirus 2; C3: complement component 3; MPO-ANCA: myeloperoxidase antineutrophil cytoplasmic antibody; ACTH: adrenocorticotropic hormone.

Parameter	Level	Reference	
White blood cells	4.7	3.5–9.1 × 10^3^/μL	
Neutrophils	49.7	44.0%–72.0%	
Lymphocytes	19.2	18.0%–59.0%	
Monocytes	12.8	0.0%–12.0%	
Eosinophils	17.3	0.0%–10.0%	
Basophils	0.6	0.0%–3.0%	
Red blood cells	4.53	3.76–5.50 × 10^6^/μL	
Hemoglobin	14.5	11.3–15.2 g/dL	
Mean corpuscular volume	99.4	79.0–100.0 fl	
Platelets	18.9	13.0–36.9 × 10^4^/μL	
Total protein	6.1	6.5–8.3 g/dL	
Albumin	3.3	3.8–5.3 g/dL	
Total bilirubin	0.7	0.2–1.2 mg/dL	
Aspartate aminotransferase	21	8–38 IU/L	
Alanine aminotransferase	11	4–43 IU/L	
Alkaline phosphatase	69	106–322 U/L	
γ-Glutamyl transpeptidase	23	<48 IU/L	
Lactate dehydrogenase	217	121–245 U/L	
Blood urea nitrogen	11.6	8–20 mg/dL	
Creatinine	1.12	0.40–1.10 mg/dL	
eGFR	51.9	>60.0 mL/min/L	
Serum Na	145	135–150 mEq/L	
Serum K	3.5	3.5–5.3 mEq/L	
Serum Cl	107	98–110 mEq/L	
Serum Ca	1.8	8.8–10.2 mg/dL	
Serum P	3.0	2.7–4.6 mg/dL	
Serum Mg	1.8	1.8–2.3 mg/dL	
Creatine kinase	64	56–244 U/L	
CRP	1.23	<0.30 mg/dL	
TSH	1.21	0.35–4.94 μIU/mL	
Free T4	0.8	0.70–1.48 ng/dL	
IgG	1073	870–1,700 mg/dL	
IgM	63	35–220 mg/dL	
IgA	237	110–410 mg/dL	
IgE	30	<170 IU/mL	
HbA1c	6.9	4.6%–6.2%	
Serum glucose	137	70–109 mg/dL	
Cholinesterase	228	234–493 IU/L	
C3	119	80–140 mg/dl	
SARS-CoV-2 antigen	Negative	Negative	
MPO-ANCA	<1.0	<3.5 U/mL	
Cortisol (early morning)	2.5	10–65 pg/mL	
ACTH	36.3	7.2–63.3 pg/mL	
TSH	1.21	0.35–4.94 µIU/mL	
pH	7.369	7.35–7.45	
PCO_2_	52.8	35–45 mmHg	
PO_2_	23.3	69–116 mmHg	
HCO_3_	30.4	22.2–28.3 mmol/L	

**Figure 1:**
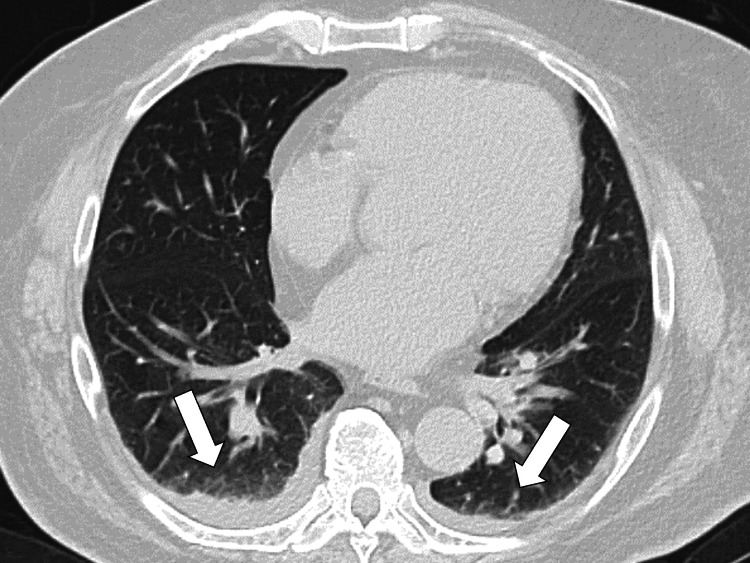
The chest computed tomography scan showed bilateral pleural effusion (white arrows).

An upper gastrointestinal endoscopy was performed to investigate anorexia and pericardial pain; however, no apparent cause was identified. Additional blood tests revealed a low early morning cortisol level of 2.5 μg/dL. An adrenocorticotropic hormone (ACTH) challenge test was performed. Cortisol levels at 30 and 60 min after the challenge were 4.7 and 6.0 μg/dL, respectively, indicating adrenal suppression and suspected adrenal insufficiency. To determine whether the adrenal insufficiency was primary or secondary, the ACTH levels were measured and found to be 36.3 pg/mL (7.2~63.3 pg/mL). In addition, the blood aldosterone level was 21.0 pg/mL (4.0~82.1 pg/mL), and renin activity was 0.9 ng/mL (0.1~2.0 ng/mL), both within reference values. Chest computed tomography (CT) revealed bilateral pleural effusions (Figure 1).

Magnetic resonance imaging of the brain revealed no abnormalities in the pituitary gland.

The patient was suspected of having hypereophilia with vasculitis because of a history of asthma, cough, and numbness in the extremities. However, the myeloperoxidase antibody proteinase 3 antibody test was negative. The patient’s itchy skin on the extremities and associated dermatitis findings led us to suspect an allergic skin disease; his blood thymus and activation-regulated chemokine level were 1,056 pg/mL (>1,000 pg/mL), indicating a severe case of adult atopic dermatitis.

The screening test was positive for the interferon gamma release assay (IGRA; T-SPOT®), showing the presence of the infection of tuberculosis. However, the patient reported no history of pulmonary tuberculosis and was considered to have a latent tuberculosis infection because of the negative triple sputum test and no nodules on chest radiography. Because adrenal tuberculosis was suspected, a contrast-enhanced CT of the abdomen was performed; however, no findings of adrenal gland calcification were observed, and the amount of pleural effusion was the same and difficult to take for further examinations. Because the occult blood in the stool was positive, a lower gastrointestinal endoscopy was performed, and multiple colonic polyps were found. However, pathological examination of the colonic polyps revealed no evidence of intestinal tuberculosis.

As no apparent secondary or tertiary cause was found, a diagnosis of primary adrenal insufficiency was made, and the patient was started on hydrocortisone 10 mg/day on admission day eight. 0.064% betamethasone ointment was used to treat atopic dermatitis. In addition, stasis dermatitis was also suspected because of venous irritation of both lower extremities and atrophy of the subcutaneous tissue of the lower legs. After initiating hydrocortisone treatment, the joint symptoms, general malaise, abdominal pain, and loss of appetite improved. On admission day 24, the patient was discharged and could perform activities of daily living.

## Discussion

We encountered a patient with primary adrenal insufficiency accompanied by latent tuberculosis. The findings of this case can be used as a basis for the diagnosis of primary adrenal insufficiency in patients with various complaints and the treatment and management of subclinical adrenal insufficiency.

Adrenal insufficiency can present with various symptoms and should always be considered in patients with such complaints. Autoimmune causes account for approximately 80% of primary adrenal insufficiency cases, while other causes include infectious diseases (tuberculosis, fungal infection, HIV), metastatic disease, and hemorrhagic infarction [7,8]. Secondary causes are most commonly tumors of the pituitary gland and surrounding areas; other causes include pituitary apoplexy and hypopituitarism [8,9]. Symptoms include fatigue, decreased appetite, weight loss, nausea, vomiting, abdominal pain, myalgia, and arthralgia. More characteristic findings of primary adrenal insufficiency include hypotension due to mineralocorticoid deficiency, salt craving, and hyperpigmentation due to ACTH overproduction [10]. Laboratory findings were characterized by hyponatremia, hyperkalemia, eosinophilia, and hypoglycemia. A diagnosis was made based on early-morning cortisol and ACTH levels. A morning cortisol level < 3 μg/dL strongly suggests adrenal insufficiency. If clinical findings suggest adrenal insufficiency but cortisol levels are ambiguous, an ACTH stimulation test is performed, and cortisol levels of 18 μg/dL or higher can rule out adrenal insufficiency [8,9]. To distinguish between primary and secondary/tertiary diseases, early-morning ACTH levels should be measured along with early-morning cortisol levels. An ACTH level twice the upper reference limit suggests the possibility of primary disease [8,9]. Treatment includes hydrocorticoid administration and continuation at the lowest dose necessary to improve symptoms.

In the present case, in which adrenal insufficiency was suspected, the ACTH level was 36.3 pg/mL, which was well within the reference level without excessive production, suggesting primary disease. However, the ACTH level was in the upper half of the reference value and considered indeterminate for establishing the deficiency level. Plasma aldosterone and renin levels were within the reference values, suggesting a central component. However, head magnetic resonance imaging showed no significant abnormalities in the pituitary gland, making the possibility of a secondary cause unlikely. Additionally, topical steroids were administered. Although the dose used is insufficient to cause adrenal insufficiency, the possibility of a tertiary nature cannot be ruled out [11]. However, aldosterone and renin may be administered if only the fascial zone of the adrenal gland is affected. Furthermore, close examination is necessary to consider the possibility of primary adrenal insufficiency [9]. Adrenal insufficiency has various presentations, as described above. In the present case, in addition to symptoms of general malaise, decreased appetite, and abdominal pain, blood tests revealed an elevated eosinophil count. When a patient presents with such symptoms, measuring cortisol levels for the differential diagnosis of adrenal insufficiency is essential. Extrapulmonary tuberculosis can also cause adrenal insufficiency; although adrenal tuberculosis is rare, accounting for only approximately 5% of active tuberculosis cases, approximately 50% of adrenal tuberculosis cases are complicated by extrapulmonary tuberculosis [12]. Therefore, other extrapulmonary tuberculosis types, such as bone, joint, and intestinal tuberculosis, should be considered while simultaneously examining both tuberculosis and adrenal tuberculosis.

Eosinophilia occurs with adrenal insufficiency, and the development of atopic dermatitis with adrenal insufficiency is considered possible. Since the eosinophil count decreased and lulled after topical steroid treatment for dermatitis, dermatitis was considered the leading cause of the eosinophilic count increase.

Latent tuberculosis infection is a state of Mycobacterium tuberculosis infection that has not developed since the first infection, and 5%-15% of cases may reactivate and develop the disease [13]. The prevalence of TB in Japan in 2021 was predicted to be 9.2 cases per 100,000 people, which is higher than that in other developed countries. The number of tuberculosis cases is expected to increase as more foreigners migrate to Japan [14,15]. The diagnosis was a latent tuberculosis infection when IGRA was positive, but chest radiography and CT were negative for active tuberculosis.

Adrenal insufficiency occurs as a direct result of glandular involvement, mainly in active tuberculosis, which leads to primary adrenal insufficiency, or Addison’s disease. In such cases, adrenal tuberculosis is often diagnosed 10-15 years after the initial infection, indicating a relatively late onset and delayed diagnosis [16]. However, the absence of adrenal enlargement or calcification does not rule out tuberculosis as the cause of adrenal insufficiency. Thus, general physicians should have comprehensive views and carefully follow-up patients in the long term through collaboration with multiple medical professionals for better quality of care [17,18].

## Conclusions

Because adrenal insufficiency can present with symptoms such as general malaise, decreased appetite, abdominal pain, and various other presentations such as elevated eosinophil count, it is essential to identify adrenal insufficiency in the differential diagnosis when examining a patient with such symptoms. In addition, since active tuberculosis is a possible cause of adrenal insufficiency, a wide range of pulmonary and extrapulmonary tuberculosis tests should be performed concurrently with tests for tuberculosis infection before starting to treat adrenal insufficiency. Adrenal insufficiency should be considered, and cortisol levels should be measured in patients presenting with general malaise and decreased appetite. Although rare, adrenal insufficiency can be caused by adrenal tuberculosis, and patients should be tested for tuberculosis and followed up continuously.
